# Exploring How Hearing, Vision, and Cognition Affect Older Adults’ Driving Exposure Patterns

**DOI:** 10.1093/geroni/igaa057.2097

**Published:** 2020-12-16

**Authors:** Jonathon Vivoda, Lisa Molnar, David Eby, Jennifer Zakrajsek, Nicole Zanier, Carolyn DiGuiseppi, Guohua Li, David Strogatz

**Affiliations:** 1 Miami University, Oxford, Ohio, United States; 2 University of Michigan, Ann Arbor, Michigan, United States; 3 Colorado School of Public Health, Aurora, Colorado, United States; 4 Mailman School of Public Health, Columbia University, New York, New York, United States; 5 Bassett Healthcare Network, Cooperstown, New York, United States

## Abstract

Better information is needed about how declines in sensory and cognitive function affect older drivers. This study assessed how hearing loss affects engagement in four challenging driving patterns. Data from the AAA Longitudinal Research on Aging Drivers study was used, including objectively-measured driving; three measures of hearing: reported hearing aid use, self-rated hearing, and the Whisper Test; visual acuity (Tumbling E); and cognition (Trail Making B). Failing the Whisper Test in both ears was related to significantly lower percentage of trips (%trips) at night, on freeways, and during rush hour, but a higher %trips >15 miles. Hearing aid use and self-rated hearing were not associated with any driving differences. Worse vision was related to a lower %trips >15 miles, while worse cognition was associated with a lower %trips at night, on freeways, and during rush hour. The Whisper Test interacted with cognition for rush hour trips.

